# Metastasis to the Bladder: A Rare Site of Recurrence of Renal Cell Carcinoma

**DOI:** 10.1155/2022/4339270

**Published:** 2022-06-17

**Authors:** Amanda Smart, Michael Wynne, Ezra Baraban, Yasser Ged, Armine Smith

**Affiliations:** ^1^Department of Urology, George Washington University Hospital, Washington, DC, USA; ^2^Department of Pathology, Johns Hopkins University School of Medicine, Baltimore, MD, USA; ^3^Department of Oncology, Johns Hopkins University School of Medicine, Baltimore, MD, USA; ^4^The James Buchanan Brady Urologic Institute and Department of Urology, Johns Hopkins University School of Medicine, Baltimore, MD, USA

## Abstract

Renal cell carcinoma (RCC) is considered to be the deadliest urologic cancer with high rates of metastasis and recurrence after nephrectomy. RCC can metastasize to nearly any organ but most commonly metastasizes to the liver, lung, brain, and bone. To date, there are only about 40 reported cases of RCC with solitary bladder metastasis. The following report contributes to this limited data set of patients with RCC who develop solitary metastasis to the bladder. A 69-year-old male presented with occasional gross hematuria and was found to have a left renal mass infiltrating the collecting system. Ureteroscopic biopsy revealed clear cell RCC, and the patient subsequently underwent radical left nephrectomy. Eight months after nephrectomy, the patient presented to the clinic with gross hematuria. In-office cystoscopy demonstrated a nodular lesion in the bladder arising from the left ureteral orifice. The patient underwent transurethral resection of the bladder mass and pathology demonstrated clear cell RCC. Subsequent imaging showed no evidence of metastatic disease. Five months after transurethral resection, the patient was found to have a left distal ureteral mass and underwent left ureterectomy with partial cystectomy. Pathology again demonstrated clear cell RCC. RCC with solitary metastasis to the bladder is rare, and there are no targeted guideline recommendations for management. Per standard of care, patients with painless hematuria and risk factors for malignancy should undergo cystoscopy. In patients with a history of RCC, metastasis to the bladder should be considered in the differential diagnosis. Patients with metastatic RCC to the bladder should undergo a thorough work-up for additional sites of metastasis. In patients with RCC who develop solitary bladder metastasis amenable to resection following nephrectomy, there is a lack of evidence to guide therapy and a multidisciplinary discussion is warranted. However, if the tumor is amenable to resection, metastasectomy is a reasonable therapeutic approach and offers the patient an improved quality of life and an opportunity for remission.

## 1. Introduction

Renal cell carcinoma (RCC) accounts for 2% of global cancer diagnoses, and about 33% of patients have metastatic disease at the time of diagnosis [1, 2]. An estimated 20-40% of patients with RCC will progress after surgical resection and develop metastases [2]. Approximately 50% of recurrences occur within 2 years of resection [3]. The lung, bone, liver, and brain are the most frequent sites of metastasis for RCC; however, metastases can be found in nearly any organ. There are about 40 cases reported, including the largest series of 11 patients with solitary metastases to the bladder [4]. Our report adds to this limited data set of patients with RCC developing a solitary metastasis to the bladder.

## 2. Case Report

**Figure 1:**
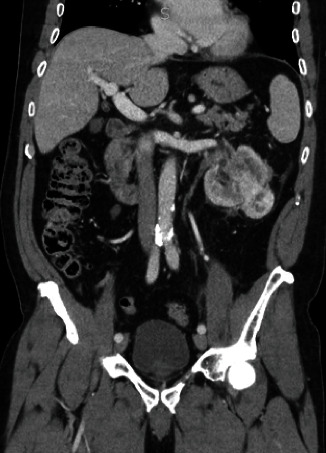
Computed tomography of renal mass demonstrating exophytic and infiltrative components of a 9 cm left interpole renal mass.

**Figure 2:**
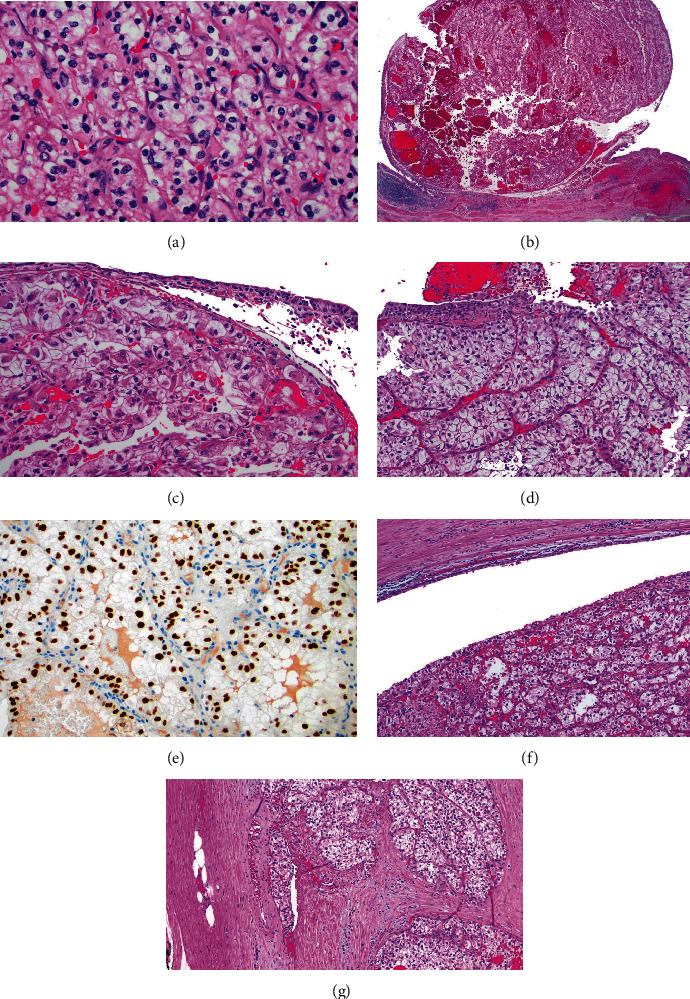
H&E-stained sections from the radical nephrectomy showed (a) areas of classic low-grade clear cell renal cell carcinoma (40x) and (b) an exophytic nodule of tumor undermining ureteral epithelium (2x). (c) Higher magnification of tumor invading the ureteral wall shows higher grade nuclei and tumor cells with voluminous and focally eosinophilic cytoplasm undermining urothelium (20x). (d) Transurethral resection of the subsequent bladder tumor showed tumor morphologically identical to that seen in the nephrectomy depicted in panel (c), undermining benign urothelium. (e) PAX8 shows diffuse nuclear labeling consistent with renal cell carcinoma metastatic to the bladder (20x). (f) Subsequent ureteral resection showed clear cell renal cell carcinoma forming an intraluminal mass with benign urothelium above (10x). (g) Focal lymphovascular invasion was noted within the ureteral wall (10x).

A 69-year-old male with hypertension, gastroesophageal reflux, and hepatitis presented with occasional gross hematuria for one month and was found to have a 9 cm left renal mass infiltrating the collecting system (Figure 1). He was a former smoker with a 12 pack-year history who quit over 30 years prior to presentation. He had no known family history of urologic malignancy; however, his father had metastatic cancer with unknown primary site. Ureteroscopic biopsy of the mass was performed and pathology demonstrated clear cell RCC, nucleolar grade 3. A laparoscopic left radical nephrectomy was performed. Intraoperatively, a portion of the adrenal gland was densely adherent to the kidney capsule, and thus, a partial adrenalectomy was also performed. Pathology demonstrated clear cell RCC nucleolar grade 3 with 10% necrosis. Histologically, the tumor showed classic areas of low-grade clear cell renal cell carcinoma, as well as higher grade areas that demonstrated invasion into renal sinus fibroadipose tissue and ureteral urothelium as well as lymphovascular invasion (Figures 2(a)–2(c)). Resection margins were uninvolved and the final pathologic stage was pT3aNx.

The patient was advised to follow up with labs and imaging every six months for the first three years and then annually. Imaging at six-month follow-up demonstrated no evidence of disease. Eight months after nephrectomy, the patient presented to the clinic with gross hematuria and was found to have a nodular bladder lesion on in-office cystoscopy. Cytology was negative for high-grade urothelial carcinoma. Transurethral resection of the bladder tumor was performed after identification of a 5 cm nodular lesion arising from the left ureteral orifice from a small stalk. Pathology demonstrated clear cell RCC (Figures 2(d) and 2(e)). Computed tomography imaging of the chest and magnetic resonance imaging of the abdomen and pelvis all demonstrated no evidence of metastatic disease. The case was discussed in genitourinary tumor board, and close follow-up was recommended with imaging every three months for one year, extending the interval from four to six months subsequently. Additionally, repeat cystoscopy was recommended if any recurrent gross hematuria developed.

**Figure 3:**
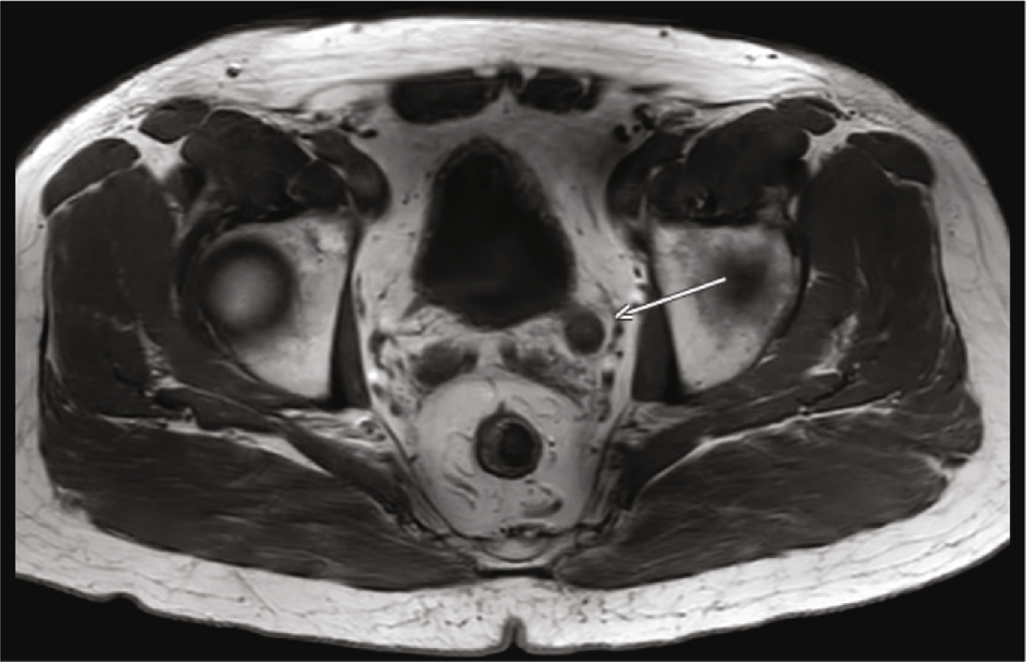
MRI with left distal ureteral recurrence (arrow).

Five months after transurethral resection of the bladder tumor, a left distal ureteral mass was identified on follow-up MRI (Figure 3). Left ureterectomy with partial cystectomy was performed and pathology again demonstrated metastatic clear cell RCC (Figures 2(f) and 2(g)).

Transcriptomic profiling and targeted gene sequencing data (BostonGene) of the primary renal tumor revealed *PBRM1* (Q167kfs∗7) loss of function (LOF) mutation, *CDKN2A* (E120∗) LOF mutation, high programmed death ligand 1(PD-L1) expression (94%), high immune active infiltrates (CD4+ T cells and B cells), and low angiogenesis signals.

## 3. Discussion

This case presents an early recurrence after nephrectomy of a metastatic RCC with a solitary metastasis to the bladder. Higher tumor stage has been found to correlate with sooner recurrence after nephrectomy with T3 tumors generally recurring between 17 and 28 months [5]. In this case, recurrence occurred after less than one year and presented in the bladder, a rare site of recurrence for RCC. The mechanism for bladder metastasis has been debated and may vary between individual patients. Proposed mechanisms include direct spread and implantation, lymphatic spread, and vascular spread especially via retrograde venous emboli through connections between the left renal vein and the bladder [6]. Direct spread to the bladder via the collecting system and ureter seems likely in this case due to involvement of the left ureteral orifice, negative nephrectomy surgical margins, and lack of other metastases although lymphangitic spread along periureteral lymphatics is also a possibility. The additional RCC recurrence in the left ureter following transurethral resection of the bladder tumor further supports this route of metastasis. In contrast, other reported cases where metastasis to the bladder has occurred along with spread to additional sites such as the brain, liver, abdomen, and mediastinum seem to arise via vascular or lymphatic spread [4]. While the liver, lung, brain, and bone remain the most frequent sites of metastasis, RCC can metastasize to a number of other rare sites including the pancreas, spleen, thyroid, vagina, rectum, and breast which further complicates our understanding of the mechanism of RCC metastasis [7–11]. An analysis of isolated RCC metastases to the pancreas supports an underlying seed-and-soil mechanism in the metastasis of RCC to rare sites [7]. Further characterization of these mechanisms in metastatic renal cell carcinoma (mRCC) is required to better understand and predict sites of metastasis. Previous studies have implicated differential microRNA expression in metastasis of RCC to different sites, and investigation of gene expression profiles in mRCC remains an interesting active area of research [12–14]. Molecular characterization of mRCC tumors, especially from rare metastatic sites such as the bladder, may aid in predicting specific sites of metastasis and contribute to improved diagnostic and therapeutic interventions. Our molecular analysis of the primary renal tumor showed low angiogenesis signal expression and higher immune signatures, which could have implications on treatment selection. Molecular characterization of the primary renal tumor and metastatic bladder lesions as part of a larger series would be helpful to understand bladder tropism in mRCC.

Treatment for oligometastatic RCC amenable to resection has long favored metastasectomy. In the more commonly observed setting of solitary pulmonary metastases, metastasectomy has demonstrated five year survival rates from 30-45% and a twofold decrease in risk of death [15]. The timing of solitary metastasis occurrence after definitive RCC treatment has been shown to play a significant role in overall survival post metastasectomy. One study reported an overall survival of 31 months if metastasis occurred before 1 year compared to 63 months if metastasis occurred after 1 year [16]. Of note, the bladder was not a site of metastasis in any of the patients included in the aforementioned study. Site of metastasis likely plays an important role in survival as demonstrated by significant differences in survival between patients with lung and bone vs. liver and brain metastasis [16, 17]. Reports of RCC metastasizing to the bladder are sparse; however, a case report from 2002 examined 27 other such cases and identified an 80% three-year survival in 13 patients with solitary metastasis to the bladder and a 20% three-year survival rate in 15 patients with multiple metastases [6]. A 2015 systematic review of 65 reported cases of RCC metastasizing to the bladder identified a 71.1% 2-year cancer-specific survival rate in 36 patients with solitary bladder metastasis compared to 25.8% in 22 patients with additional metastases. The same review identified a 2-year cancer-specific survival of 34.6% in patients who developed bladder metastasis within 1 year of primary RCC diagnosis compared to 58.4% after 1 year of diagnosis. All bladder lesions in these cases were managed surgically [18]. In addition to reported data on timing and site of metastasis, use of risk stratification tools such as the International Metastatic Renal Cell Carcinoma Database Consortium criteria and Memorial Sloan Kettering Cancer Center criteria plays an important role in predicting prognosis in patients with mRCC.

Guidelines uniformly recommend metastasectomy for resectable single site RCC metastases [19]. Until recently, there was no role for postmetastasectomy adjuvant therapy as neither sorafenib nor pazopanib have demonstrated recurrence-free survival benefit in patients with mRCC [20, 21]. However, a recent phase III trial demonstrates improved disease-free survival with adjuvant pembrolizumab therapy following nephrectomy and metastasectomy in patients with oligometastatic RCC [22]. Due to the rare occurrence of metastatic RCC to the bladder, there are no targeted guideline recommendations for management. The limited data that is currently available suggests that postnephrectomy metastasectomy can afford a favorable prognosis, especially if the bladder is the sole site of metastasis as with the patient reported in this case. However, appearance of bladder metastasis sooner than one year postnephrectomy may be associated with worse survival, which complicates the prognosis of this patient. This patient's prognosis is further complicated by the additional ureteral recurrence following transurethral resection of the bladder tumor. While urothelial carcinomas comprise the vast majority of malignant bladder tumors, this case reinforces that in a patient with a history of RCC, discovery of a bladder tumor on cystoscopy should raise clinical suspicion for RCC recurrence. Additionally, in patients who develop metastatic RCC to the bladder, thorough work-up for additional sites of metastasis is essential for determining prognosis as well as the need for systemic therapy.
